# 

**Published:** 1903-04-15

**Authors:** 


### CONTENTS.

Clinics at the Ohio State Meeting - - - - - 163

The Function and Value of the Dental Society,

*By N. S. Hoff, D.D.S.* 171

Dental Education, *By C. N. Johnson, L.D.S., D.D.S.* 179

Italian Dental Curriculum, *By Dr. Vincenzo Guerini* 187

The Manipulation and Care of Cements,
*By W. V-B. Ames, D.D.S.* 190

Preliminary Examinations of the Teeth,
*By E. A. Bogue, M.D.* 194

A Plea for More General Knowledge of Dental Text-books 196

The Professional Spirit,
*By C. N. Johnson, L.D.S., D.D.S.* 198

Editorial, - - - - - - - - - - - 207

Bibliography, - - - - - - - - 208

Announcements, - - - - - - - 209

Indents, - - - - - - - - - - - 212

